# Antioxidant and pro-angiogenic effects of corilagin in rat cerebral ischemia via Nrf2 activation

**DOI:** 10.18632/oncotarget.22023

**Published:** 2017-10-24

**Authors:** Yi Ding, Danjun Ren, Hang Xu, Wenxing Liu, Tianlong Liu, Liang Li, Jianguang Li, Yuwen Li, AiDong Wen

**Affiliations:** ^1^ Department of Pharmacy, Xijing Hospital, The Fourth Military Medical University, Xi’an 710032, China; ^2^ XinJiang Medical University, Wulumuqi 830001, China

**Keywords:** corilagin, Nrf2, oxidative stress, angiogenesis, ischemic stroke

## Abstract

The nuclear factor erythroid-2-related factor 2 (Nrf2) pathway has been considered as a potential target for neuroprotection in stroke. The aim of present study was to determine whether corilagin, a novel Nrf2 activator, can protect against ischemia-reperfusion injury and explore the underlying mechanism involved. *In vivo*, rats exposed to middle cerebral artery occlusion were applied to establish an ischemic stroke model. Posttreatment of corilagin significantly reduced infarct volumes and apoptotic cells as well as improved neurologic score after reperfusion, together with increased vascular density in the ischemic penumbra. Meanwhile, posttreatment with corilagin in MCAO rats significantly decreased malondialdehyde levels, restored the superoxide dismutase and glutathione activity, elevating the Nrf2, heme oxygenase-1, the vascular endothelial growth factor (VEGF) and VEGF receptor 2 (VEGFR2) expression. However, consecutive intrathecal injection of short interference RNAs targeting Nrf2 at 24-h intervals 72 h before ischemia reduced the beneficial effects of corilagin. In primary cultured neurons, corilagin dose-dependently protected against oxygen and glucose deprivation-induced insult, but the protective effect of corilagin was attenuated by knockdown of Nrf2. In conclusion, these findings indicate that corilagin exerts protective effects against cerebral ischemic injury by attenuating oxidative stress and enhancing angiogenesis *via* activation of Nrf2 signaling pathway.

## INTRODUCTION

Ischemic stroke is one of the leading causes of morbidity and mortality [1]. Ischemic stroke is caused by reduced blood flow into the brain, resulting in damage to the brain tissues. Tissue plasminogen activator remains the only FDA-approved treatment for ischemic stroke, but benefits only 3-8.5% of stroke patients due to the narrow time window and adverse effects [1]. It is imperative to find novel therapeutic strategies for stroke.

Nrf2 related pathways have been considered molecular targets in pharmacologic intervention for ischemic stroke [2]. Nrf2 is necessary for the induction of a significant set of antioxidant genes that act in synergy to remove reactive oxygen and nitrogen species (ROS/RNS) through sequential enzymatic reactions. Beneficial actions of the modulating Nrf2 signaling pathway in stroke, such as improving neurological function, anti-oxidative and anti-apoptotic effects, have been noted [3]. Consistently, in our previous studies, we have demonstrated that Nrf2 activators can decrease oxidative stress and have protective roles in models of stroke [4–6]. On the other hand, the angiogenic effect of Nrf2 was identified in recent years. Nrf2 also induces the expression of hemo oxygenase-1 (HO-1) [7], which has been proved to increase VEGF expression and promote angiogenesis [8, 9]. For example, study of epigallocatechin-3-gallate for ischaemic stroke also indicates that Nrf2 plays a positive role in angiogenesis by up-regulating VEGF [10].

**Figure 1:**
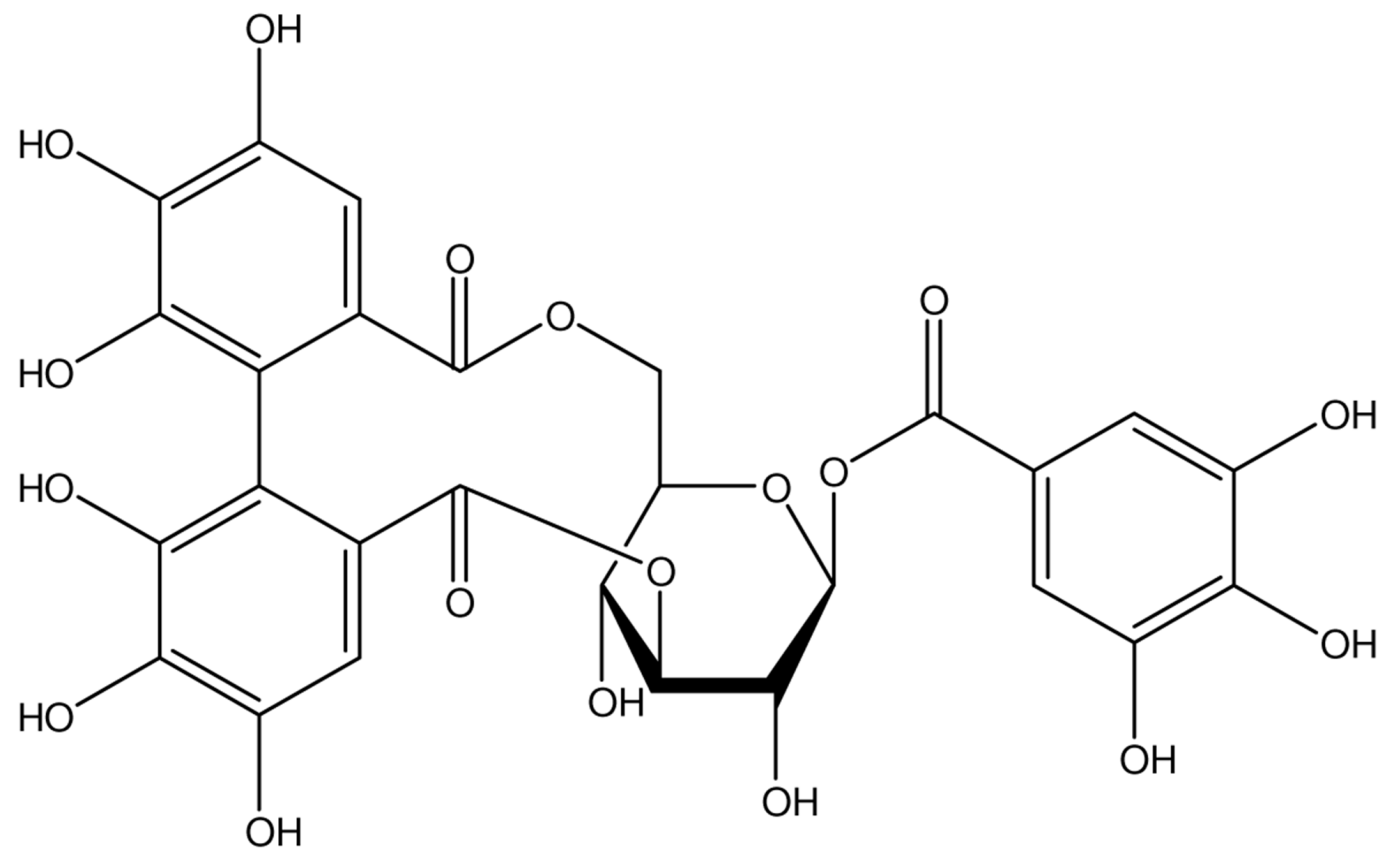
Chemical structure of CL

Epidemiological evidence suggests that polyphenol compounds play an important role in prevention and treatment of cardiovascular diseases mediated by the stimulation of Nrf2 [11, 12]. Corilagin (CL), being given in Figure 1, the major constituent of *Terminalia chebula* and *Phyllanthus emblica*, is a phenolic compound with various medicinal potentials including antioxidative [13], anti-inflammatory [14], and antihypertensive effects [13], as exhibited by experiments with different animal models. Especially, recent researches indicated that extracts from *Terminalia chebula* ameliorate infarct volume, improve neural function and increase brain microvessels density in cerebral infraction rat [15]. The effect of extracts from *Terminalia chebula* on activation of the Nrf2/HO-1 antioxidant pathway has also been reported [16, 17].

Considering the beneficial properties of *Terminalia chebula* in stroke and the possible role of the Nrf2 pathway, we hypothesized that CL is neurovascular-protective and improves stroke outcome via the Nrf2 pathway. In the present study, we aimed to investigate the effect of posttreatment with CL on ischemia–reperfusion (IR) injury and functional outcome after 7 days in a middle cerebral artery occlusion (MCAO) model, and elucidate the potential roles for Nrf2 activation in the antioxidant and pro-angiogenic effect of CL.

## RESULTS

### CL alleviated cerebral ischemic injury

**Figure 2:**
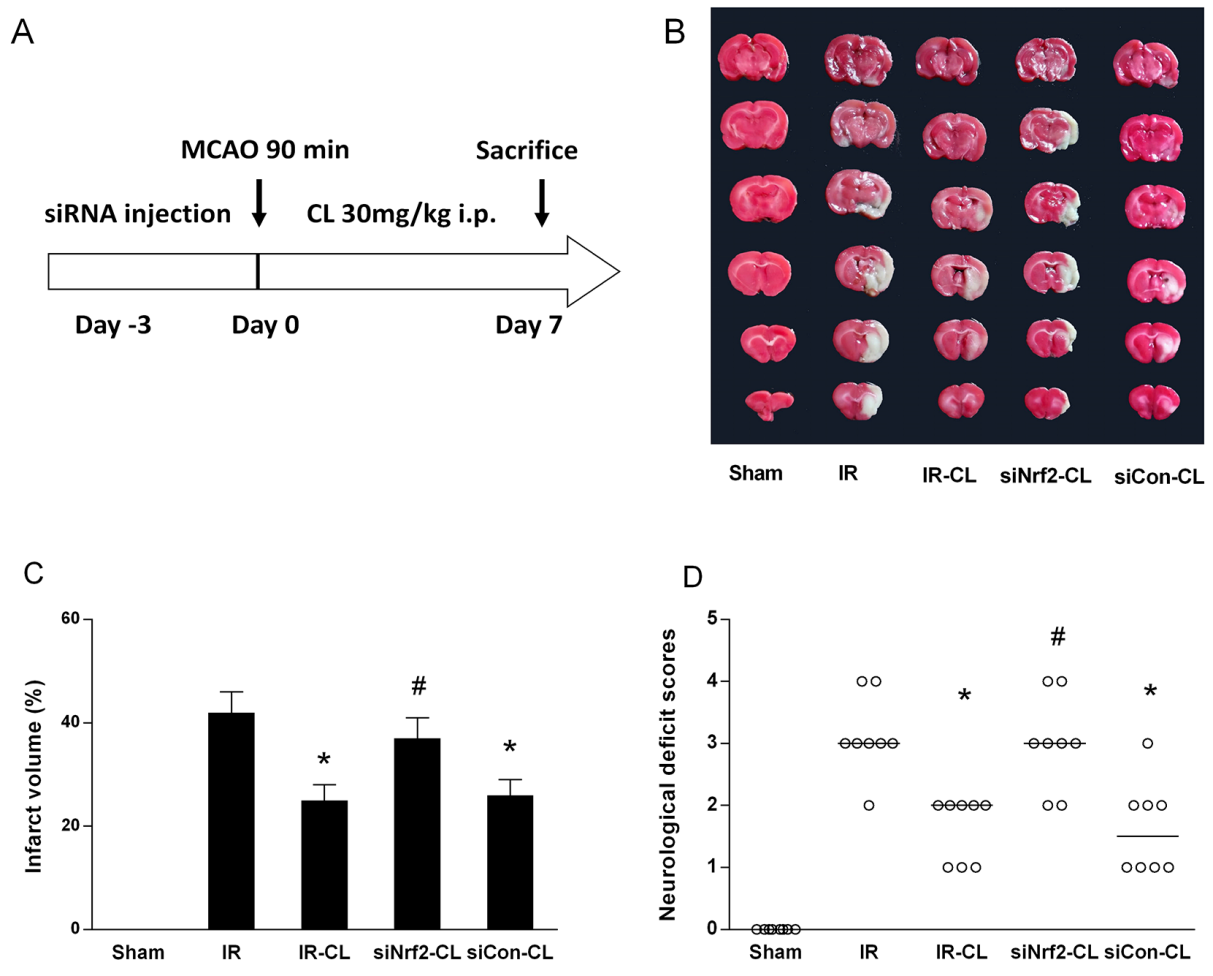
CL reduce brain injury and improves neurological outcome These neuroprotective effect was abolished by Nrf2-siRNA. **(A)** Diagram of the experimental protocols. **(B)** Photographs of rat brain TTC staining. **(C)** Quantitative analysis of infarct size. Values are mean ± SD (n = 8). **(D)** Neurological scores of animals in different groups. Data are presented as the median (range) (n = 8). ^*^ P < 0.05 vs. IR group; ^#^ P < 0.05 vs. IR-CL group.

The infarct volume of the ipsilateral brain was measured with 2,3,5-triphenyltetrazolium chloride (TTC) staining. CL significantly decreased infarct volumes 7 days after reperfusion (Figure 2B and 2C). As shown in Figure 2C, CL treatment could statistically reduce the infarct volume from 42.3±4.2% (IR group) to 25.1± 3.3% (IR-CL group) (n=8 animals per group; P<0.05). In terms of evaluation of neurological function, neurological deficit grading system was carried out. For the rats in the IR group, they remained with the highest neurological deficit score. CL treatment significantly reduced the neurological deficit score compared with the vehicle treatment (Figure 2D) (n=8 animals per group; P<0.05). While these effects were abolished by intrathecal Nrf2 small interfering RNA (siRNA) pretreatment in siNrf2-CL group (Figure2B-2D), and control siRNA had no effect (P>0.05 vs. IR-CL).

**Figure 3:**
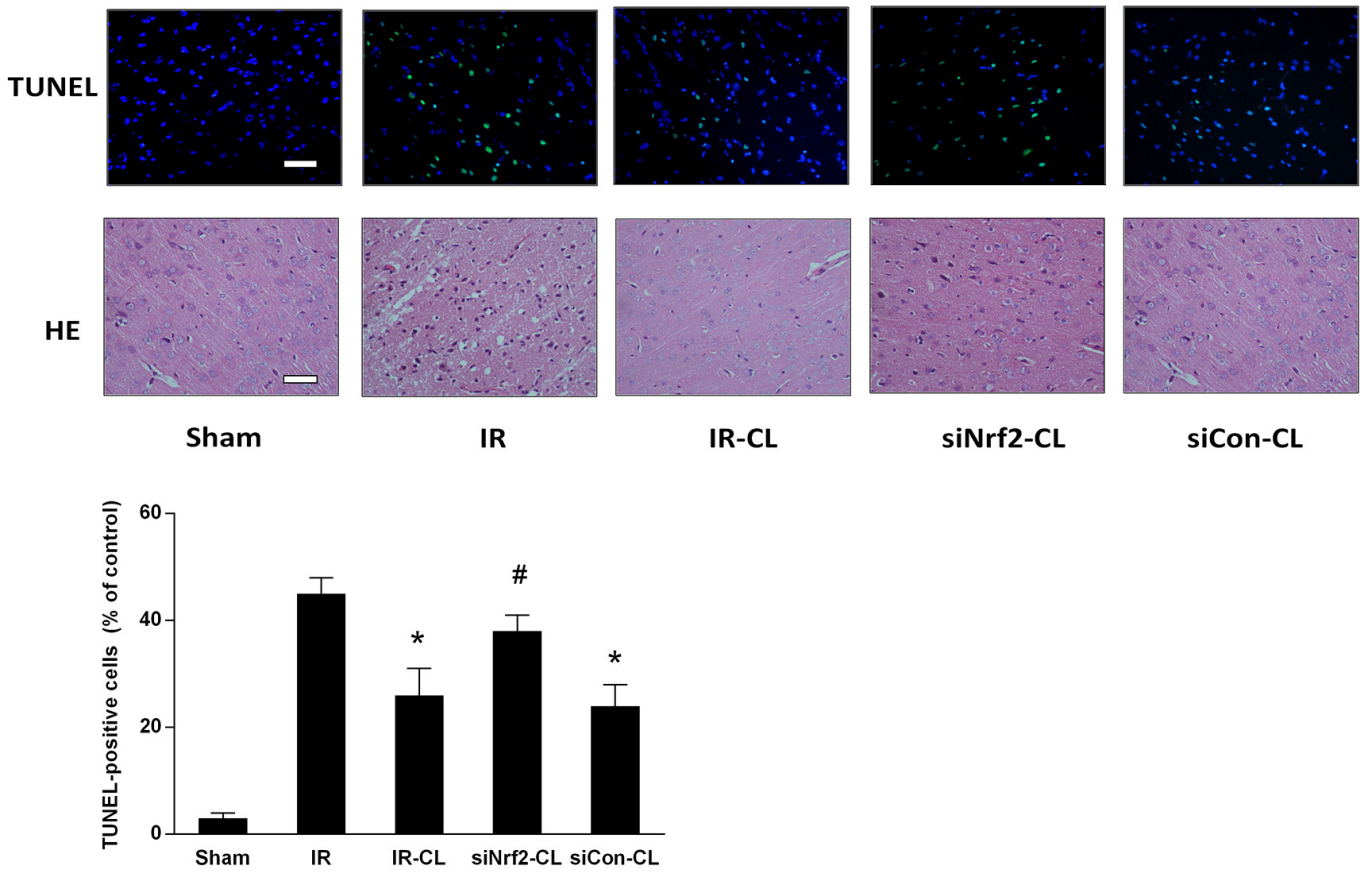
CL alleviates neuronal death at 7 days after MCAO Representative images of HE staining and TUNEL staining performed on sections from ischemic cortex at 7 days after MCAO in rats (TUNEL-positive cells in green, DAPI in blue; Scale bar = 40 μm). Quantitative analysis of TUNEL-positive cells is also exhibited. ^*^ P < 0.05 vs. IR group; ^#^ P < 0.05 vs. IR-CL group. Values are mean ± SD (n = 8).

Furthermore, the protective effect of CL against cerebral ischemic damage was confirmed by Hematoxylin and eosin (HE) staining and dUTP-digoxigenin nick end labeling (TUNEL) staining on sections from ischemic cortex at 7 days after IR (Figure 3). In IR group, the cells were arranged irregularly in ischemic peri-infarct of cerebral cortex and the number of TUNEL-positive cells was increased compared with sham group. More intact neurons with fine granular cytoplasm in CL-treated IR rats. The percentage of TUNEL-positive cells in the ischemic cortex was decreased from 45.0±3.2 to 26.5±4.4 % by CL treatment in IR-CL group (P<0.05). However, these protective effects were abolished by intrathecal Nrf2 siRNA pretreatment in siNrf2-CL group (P<0.05). No statistic difference was found between IR and siNrf2-CL group (P>0.05). These results suggest that CL alleviates the neurological damage caused by IR injury and that the effect is Nrf2-dependent.

### CL attenuated oxidative stress

The results of estimation of biochemical parameters were shown in Table 1. The superoxide dismutase (SOD) and glutathione peroxidase (GPx) activity in the cortex was decreased in the IR group compared with the sham group, which was restored by CL (n=8 animals per group; P <0.05). The malondialdehyde (MDA) level in the cortex, which is an index of lipid peroxidation, was significantly increased in vehicle group compared with the sham-operated group. An evident reduction of the MDA level was observed in IR-CL group compared with IR group (n=8 animals per group; P<0.05). Blocking Nrf2 signaling pathway by Nrf2 siRNA abolished the antioxidative effects of CL treatment.

**Table 1:** Levels of SOD, GPx, and MDA in the cortex at 7 days after MCAO in each group. Data are expressed as means ± SD (n=8)

Groups	Sod U/mg	GPx U/mg	MDA μmol/mg
Sham	134.5 ± 7.3	85.3 ± 5.5	5.5 ± 1.2
IR	93.6 ± 7.1	44.6 ± 4.4	22.3 ± 2.8
IR-CL	112.9 ± 10.1^*^	74.3 ± 5.7^*^	14.3 ± 1.2^*^
siNrf2-CL	94.3 ± 9.7^#^	52.8 ± 7.5^#^	18.7 ± 2.2^#^
siCon-CL	111.2 ± 9.8^*^	77.1±9.4^*^	15.5 ± 1.1^*^

^*^ P < 0.05 vs. IR group; ^#^ P < 0.05 vs. IR-CL group.

### CL promoted angiogenesis in ischemic cortical tissue

**Figure 4:**
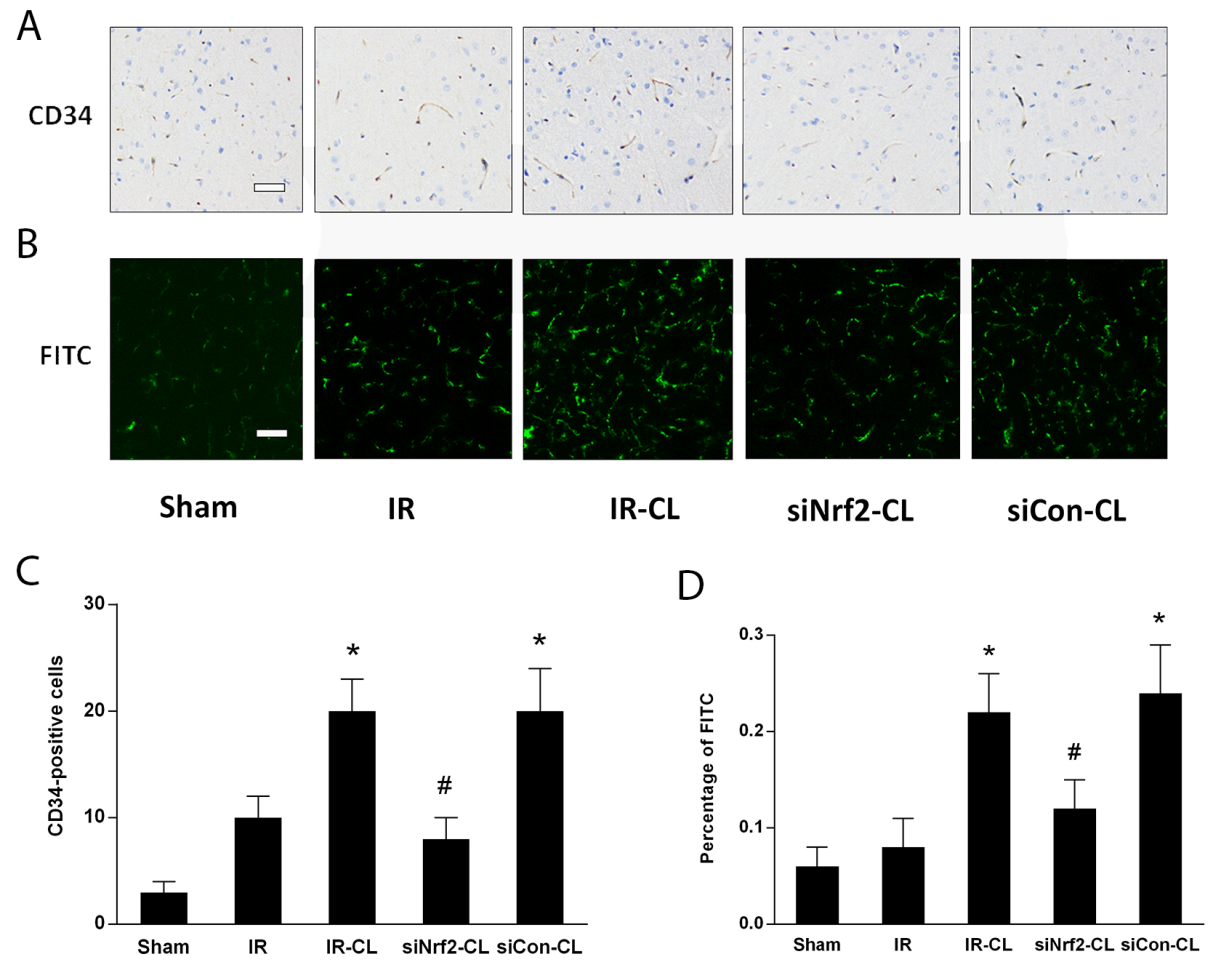
CL promotes angiogenesis after stroke Immunohistochemistry staining of CD34 **(A)** and FITC **(B)** in the peri-infarction area of rats on day 7 after MCAO. Scale bar = 40 μm. Quantitative analysis of percentage of CD34 **(C)** and FITC **(D)**. P < 0.05 vs. IR group; ^#^ P < 0.05 vs. IR-CL group. Values are mean ± SD (n = 8).

In this study, to evaluate the effect of CL on ischemic brain microcirculation, we examined microvessels perfused with FITC-dextran and the expression of the endothelial cell marker CD34 in ischemic ipsilateral cortical tissue (Figure 4). CD34 and FITC-dextran colocalizationed vessels density was significantly higher in the IR-CL group than in the IR group at 7 days after surgery. However, siNrf2 RNA treatment could deteriorate this phenomenon. Quantification of FITC and CD34 percentage confirmed these observations.

### CL increased the expression of Nrf2 and HO-1

**Figure 5:**
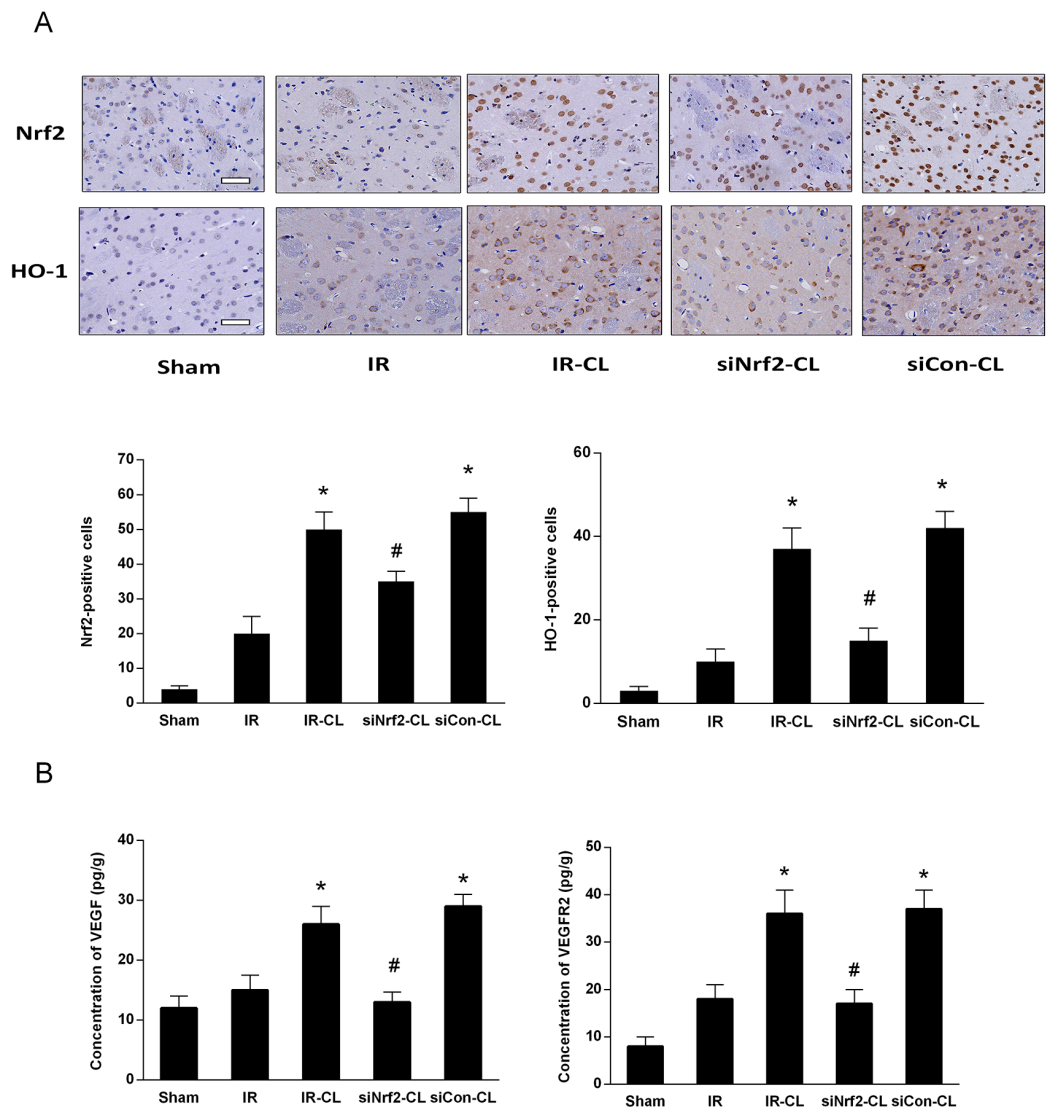
CL upregulates Nrf2/HO-1 and VEGF/VEGFR2 signaling pathway after stroke **(A)** Immunofluorescence staining of Nrf2 and HO-1 in the peri-infarction area of rats on day 7 after MCAO. Scale bar=40 μm. **(B)** ELISA analysis of VEGF and VEGFR2 levels in the ischemic brain on day 7 after MCAO. ^*^ P < 0.05 vs. IR group; ^#^ P < 0.05 vs. IR-CL group. Values are mean ± SD (n = 8).

**Figure 6:**
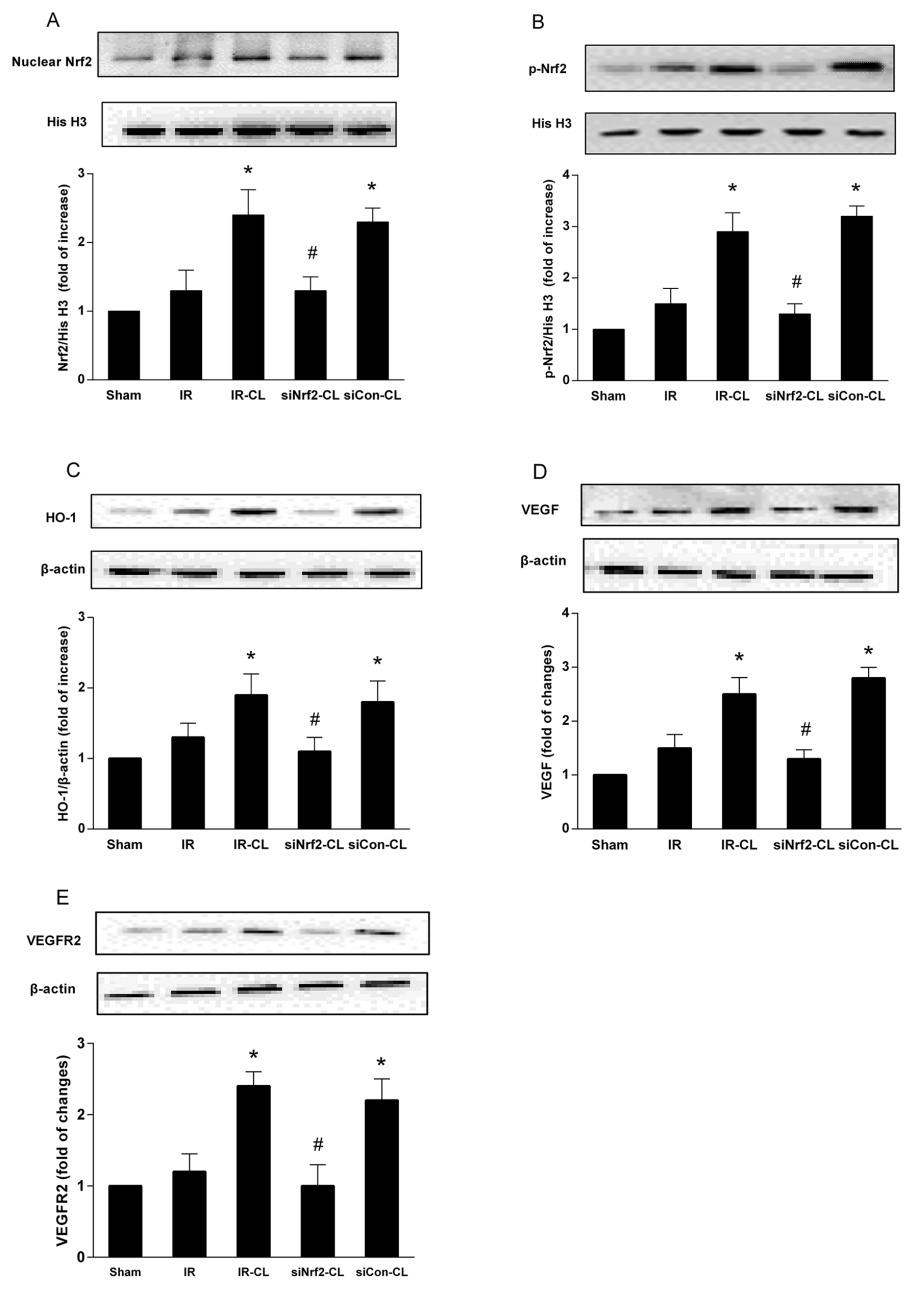
Western blot analysis of proteins CL significantly enhanced the expression of Nrf2 **(A)**, p-Nrf2 **(B)**, HO-1 **(C)**, VEGF **(D)** and VEGFR2 **(E)** in peri-infarct cortex after stroke, and this effect was abolished by Nrf2-siRNA. ^*^ P < 0.05 vs. IR group; ^#^ P < 0.05 vs. IR-CL group. Values shown are percent of ratio and are expressed as mean ± SD (n = 8).

To identify whether Nrf2 signaling is involved in the neuroprotective effect of CL, we analyzed ischemic brain tissue by western blot and immunohistochemistry staining. Immunohistochemistry staining showed that on day 7 after surgery CL treatment significantly increased the number of Nrf2-and HO-1 positive cells in the cortex (Figure 5). In sham group, few cells were stained by Nrf2 and HO-1. Western blot analysis of cortical tissues at 7 days after MCAO showed that protein expression of nuclear Nrf2 and total HO-1 was increased, and this effect was enhanced by CL (Figure 6A, and 6C). However, both of western blot and immunohistochemistry staining results showed that the elevated expression of Nrf2 induced by CL was inhibited by Nrf2 siRNA.

### CL increased the expression level of VEGF and VEGFR2 via Nrf2

Enzyme-linked immunosorbent assay (ELISA) test and western blot analysis showed that MCAO rats had higher expression of VEGF and VEGFR2 in the ischemic brain than that of IR group at 7 days after IR (Figure 6D and 6E). CL treatment significantly enhanced the stroke-induced upregulation of VEGF signaling pathway (P<0.05), and these effects were abolished by intrathecal Nrf2 siRNA.

### CL suppressed loss of cell viability via Nrf2

**Figure 7:**
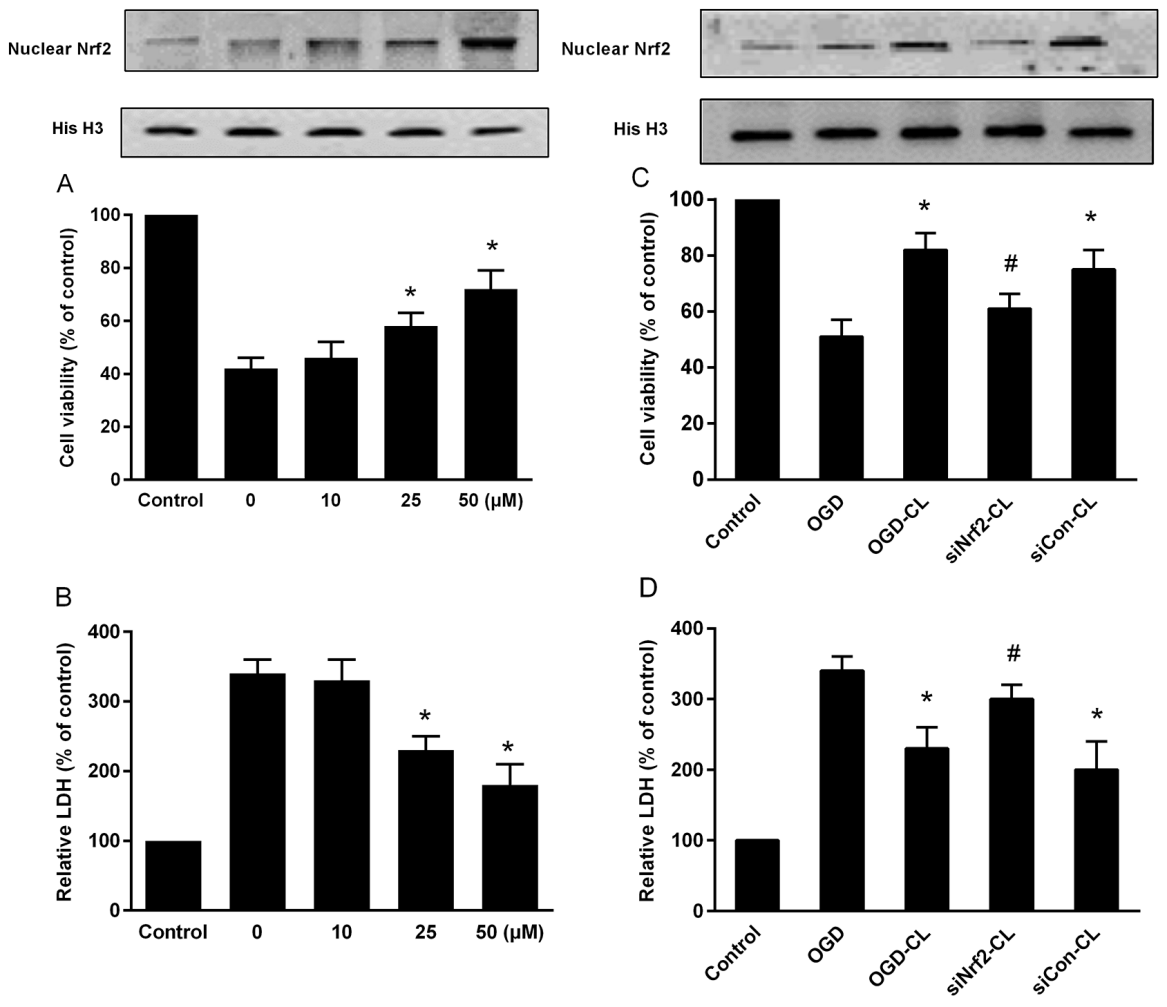
Effect of CL on cell viability and LDH release in primary culture of rat cortical neurons exposed to OGD **(A)** The cell viability. **(B)** LDH release. **(C)** Cells were treated for 48 h with control or siRNA, then subjected to 60 min OGD followed by the MTT assay; **(D)** LDH release. The control group (normal cell group) was defined as 100%. ^*^ P < 0.05 vs. OGD group; ^#^ P < 0.05 vs. OGD-CL group. Data represent means ± SD of triplicate independent experiments.

As shown in OGD treatment resulted in 58% cell death (P<0.05). CL significantly blocked OGD-induced cell death concentration dependently (P<0.05; Figure 7A). Compared to the OGD group, the viability of the cells treated with 30 μM CL was increased by approximately 30% (P<0.05). CL treatment decreased OGD-induced LDH release (P<0.05) (Figure 7B). We also found that CL increased Nrf2 expression in a concentration dependent manner (Figure 7A).

To confirm the role of Nrf2 in CL-mediated neuroprotection, we transfected control (si-control), or Nrf2-specific (si-Nrf2) siRNA in primary cultured cortical neuron for 48 h. Knockdown of Nrf2 inhibited cell viability that was increased by CL under OGD (Figure 7C). In consistent, knockdown of Nrf2 decreased LDH level compared with the si-control group (Figure 7D). Taken together, CL effectively prevented neuron from OGD damage involved activation of Nrf2 pathway.

## DISCUSSION

Dietary and plant polyphenols exert neuroprotective effects and improve neurological recovery in cerebral ischemia by antioxidant and pro-angiogenic effect [18–20]. Our research for the first time shows that CL, a kind of phenolics, improve neurologic scores, reduced infarct volume, ameliorated neuronal apoptosis and oxidative damage, and promoted angiogenesis in MCAO model. Moreover, we also found that CL-enhanced neuroprotection and VEGF expression was mediated by Nrf2 activated. These data suggest that specific polyphenols could be beneficial for the treatment of ischemic stroke associated with oxidative stress and angiogenesis via modulating Nrf2.

After ischemic stroke, the highly concentrated ROS would damage the brain tissue and be detrimental to the angiogenesis. Under normal physiological conditions, Nrf2 combines with the regulatory kelch-like erythroid cell-derived protein with CNC homology associated protein 1 (Keap1) in the cytoplasm. Oxidative stress induces Nrf2 phosphorylation, activation and nuclear accumulation of Nrf2, which upregulates endogenous antioxidant defence to restore cellular redox homeostasis [3, 21]. Animal studies have shown that antioxidant treatment in stroke can reduce brain damage and promote angiogenesis [22, 23]. Consistently, immunohistochemistry staining and western blot analysis in present study revealed that the expression of nuclear Nrf2, p-Nrf2 and HO-1 was significantly higher in IR-CL and siCon-CL groups than in the IR group. Furthermore, Nrf2 knockdown by siRNA inhibited the expression of Nrf2 and HO-1, and obviously eliminated the neuroprotective effects of CL. In our study on OGD model, data obtained from neuronal cells transfected with Nrf2-siRNA confirmed that Nrf2 played a fundamental role in neuroprotection induced by CL. Similarly, existing data has demonstrated that Nrf2-deficient mice demonstrated more severe neurologic deficits after intracerebral hemorrhage [24]. Another study on wild type and Nrf2-deficient mice also showed that Nrf2 reduces ischemic brain injury by protecting against oxidative stress [25].

SOD and GPx dismutates superoxide to hydrogen peroxide and oxygen [26]. MDA are produced by both oxidative stress-induced peroxidation and enzymatically produced lipid peroxidation during the arachidonic acid cascade which is an important element of post-ischemic secondary injury [26]. We find enhanced activity of SOD and GPx with a decreased level of MDA with CL treatment in IR-CL and siCon-CL groups. This was reversed by pretreatment of Nrf2 siRNA in siNrf2-CL group, indicating that therapeutic effect of CL is related to enhancing Nrf2 regulation and therefore alleviates oxidative stress in MCAO rats. In fact, upregulation of Nrf2 has been proved to be the key mechanism of decrease oxidative stress and promote long-term neurologic functions in stroke [27].

The role of Nrf2 in angiogenic effect was identified in recent years [28]. Post-ischemic angiogenesis greatly improves the recovery of tissue perfusion, supporting the survival of neurons and neural progenitor cells and promoting long-term functional recovery. Except for scavenging free radical to creat a better microenvironment for brain repair, Nrf2 also induces the expression of HO-1, which has been proved to increase VEGF expression and promote angiogenesis [29, 30]. Studies of atherosclerosis and multiple sclerosis also indicate that Nrf2 plays a positive role in angiogenesis by upregulating VEGF-VEGFR2 signaling pathway [8, 31]. VEGF is a key signaling molecule involved in the formation and stabilization of blood vessels and participated in the angiogenesis after brain ischemia [32]. And VEGFR2 activity mediates the pro-angiogenic effect of VEGF, and the downstream molecule plays an essential role in endothelial cell proliferation and survival [33].

Our results showed that the number of newly generated vascular endothelial cells was higher after MCAO, indicating that treatment with CL for 7 days after ischemia significantly increased the number of vessels in the peri-infarct areas. To explore the underlying mechanism by which CL promotes angiogenesis, we evaluated the expression of angiogenic factors VEGF and VEGFR2. Notably, we found that compared to IR group, MCAO rats treated CL in IR-CL and siCon-CL groups had more proliferative vessels, higher expression of VEGF and VEGFR2 in the peri-infarction area, partially confirming that CL play its neuroprotective roles more than by activating anti-oxidative pathway.

*In vitro* as stated by the MTT assay and LDH measurement, treatment with CL before OGD damage can significantly reduce cell death and increase Nrf2 expression in concentration-related manner. Another key finding of the current study was that knockdown of Nrf2 in primary cultured cortical neurons that were subjected to OGD partly diminished neuroprotective effect of CL. These *in vitro* data support our *in vivo* results and show that CL plays a neuroprotective effects against cerebral ischemic injury by Nrf2 signaling.

In the light of current knowledge, this is the first demonstration of the anti-oxidative and pro-angiogenic potential of CL on IR injury in rats. Moreover, CL induced a neuroprotection against IR *in vivo* and protected against OGD-induced cell death by activating the Nrf2. Notably, VEGF might be the downstream regulator of Nrf2 in the mediation of CL-induced neuroprotection. Nrf2 phosphorylation and translocation may be regulated through several signal transduction pathways such as MAPK and Akt pathways [34, 35]. Further investigations are needed to elucidate the detailed signaling cascades involved in the regulation of Nrf2/VEGF. In conclusion, our findings suggest that CL has neuroprotective effects against cerebral ischemic injury.

## MATERIALS AND METHODS

### Animals

All procedures involving animals were approved by the Institutional Animal Care and Use Committee of the Fourth Military Medical University and were performed in accordance with published National Institutes of Health guidelines. Sprague-Dawley rats, weighing 280-320 g, were obtained from the animal center of Fourth Military Medical University. Rats were housed in a temperature- and humidity-controlled room for at least 1 week before surgery. Standard animal chow and water were freely available, and all efforts were made to minimize animal suffering.

### Transfection of siRNA in rat brain

Nrf2 siRNA (5′-CAG CAG AAC UGU ACC UGU UU-3′ and 5′- ACC AGG UAC AGU UCU GCU GUU-3′) and control siRNA (5′-UAG CGA CUA AAC ACA UCA AUU-3′ and 5′-UUG AUG UGU UUA GUC GCU AUU-3′) were provided by GenePharma Corporation, Shanghai, China. Scramble siRNA was dissolved in RNase-free water (2 μg/μl. Rats were anesthetized with 3.5% chloral hydrate (350 mg/kg, ip) and placed in a stereotaxic apparatus (Taimeng Software, Chengdu, China). siRNA (10 μl) was injected ipsilaterally into the left lateralcerebral ventricle at 24 h interval for 3 days prior to ischemia or sham surgery. siRNA was slowly injected into the left lateral ventricle over a 20-min duration using a Hamilton microsyringe with the coordinates of 1.0 mm posterior to the bregma, 2.0 mm lateral to the midline, and 4.0 mm ventral to the surface of the skull under the guidance of a stereotaxic instrument. Knockdown of Nrf2 was assessed by Western blot assay (Supplementary Figure 1).

### Drug treatments

CL (purity 99%) was obtained from the China National Institute for the Control of Pharmaceutical and Biological Products. Figure 2 shows the study design. Rats were divided randomly into the following five groups using a random number table generated by SPSS 18.0 (SPSS Inc., Chicago, IL, USA): Sham, IR, IR-CL, siNrf2-CL and siCon-CL group. Sham animals were subjected to surgery but no IR injury. The vehicle control groups of ischemic and sham-operated rats received an equal volume of normal saline. IR rats underwent 90 min of ischemia and subsequent reperfusion but without additional treatment. CL diluted with saline (30 mg/kg) was administered to rats by intraeritoneal injection once daily starting at 3 h after MCAO for 7 consecutive days. The dose was selected according to the published study [14], which could reach to physiological concentrations in brain. In our preliminary experiment, different doses of CL (10, 30 and 70 mg/kg) were used, and the dose of 50 mg/kg significantly reduced rats infarct volumes and improved neurological deficit scores, but there is no difference between 30 mg/kg and 70 mg/kg (Supplementary Figure 2).

### Middle cerebral artery occlusion model

MCAO model was performed as reported previously [36]. Focal ischemic infarction was produced in the right middle cerebral artery territory. Rats were anesthetized using 2.0 to 3.0% isoflurane and maintained using 1.0 to 1.5% isoflurane (both in 70% N_2_O/30% O_2_). At 2 h after the induction of ischemia, the filament was slowly withdrawn. The neck incision was closed and rats were allowed to recover. The animals were allowed to survive for 7 days with free access to water and food. At the end of the reperfusion period, animals were euthanized with ketamine/xylazine (150:10 mg/kg) mixture, and then intracardially perfused with 250 ml of saline followed by 300 ml of 4% paraformaldehyde. Mean arterial blood pressure, pH, arterial blood gases, and blood glucose levels during treatment were evaluated. Between groups, no significant differences in physiological variables (Supplementary Table 1).

### Measurement of neurological score and infarct size

Neurological scores were evaluated 7 days after the MCAO using a 5-point scoring system was to evaluate the neurologic deficits in rats [36]. Longa score was used to determine motor motion functions, as follows: 0, no deficits; 1, difficulty in fully extending the contralateral forelimb; 2, unable to extend the contralateral forelimb; 3, mild circling to the contralateral side; and 4, severe circling. 0 or 4 were excluded from the study. The higher the score, the more severe the injury. Scoring of each rat was performed within 1 min and repeated three times.

Animals were carried with neurological scoring at 7 days after MCAO followed by euthanization, then brains were harvested and sectioned into 2.0 mm coronal sections. Sections were subjected to TTC staining. The percentage of TTC-stained tissue with respect to the whole piece of tissue was analyzed by Image J software (NIH, USA).

### HE and TUNEL staining

At 7 days after MCAO, HE staining was performed to show the morphological features of injured neurons in cerebral cortex. TUNEL staining was performed on paraffin-embedded sections. Commercially available reagents (Promega, DeadEnd Flurometric Tunel System) were used to perform TUNEL analysis. The total number of TUNEL positive cells in the ipsilateral hemisphere was counted in five different fields for each section by an investigator who was blinded to the studies by a fluorescent microscope (Olympus, Tokyo, Japan).

### Assessment of oxidative stress

The right cerebral cortex tissue was homogenized in 2 ml of 10 mM phosphate buffer (pH 7.4). After centrifugation at 12,000 g for 20 min, the SOD, GPx and MDA content in the supernatant were assessed spectrophotometrically with the corresponding kits (Nanjing Jiancheng Biochemistry Co., Nanjing, China). The protein concentrations were determined by the Bradford method.

### Measurement of microvascular patency

Following administration of abdominal anesthesia using 10% chloral hydrate (0.3 ml/100 g), rats were injected with 1 ml of 50 mg/ml fluorescein isothiocyanate (FITC)-dextran via the femoral vein 7 days after MCAO. After 1 min, the rats were euthanized to isolate brain tissues in the cerebral ischemic penumbra. Tissues were fixed for 48 h by instillation of 4% paraformaldehyde-PBS and sliced into 20-μm-thick coronal sections. Three coronal cross sections (20 mm) were analyzed using confocal microscope (Nikon, Japan). Data are presented as the number of FITC pixels divided by the total number of pixels within the field of view, expressed as a percentage.

### Immunohistochemistry staining

Eight rats in each group were deeply anesthetized at 24 h after injury. Then, brain tissues were rapidly removed and immersed in 4% paraformaldehyde for 72 h. Slices were incubated with 3% H_2_O_2_ at 37°C for 10 min, normal goat serum for 30 min at room temperature, and primary antibodies of interest at 4°C overnight, and then rinsed three times with 0.1 M phosphate-buffered saline (PBS). Rabbit anti-rat CD34 polyclonal antibody (1:200, Santa Cruz Biotechnology, USA), rabbit anti-rat Nrf2 polyclonal antibody (1:200, Santa Cruz Biotechnology, USA), and goat anti-rat HO-1 polyclonal anti-body (1:200, Santa Cruz Biotechnology, USA) were used to detect protein expression. Then, the slices were incubated with secondary antibodies. Hematoxylin staining was selected as the counterstain. An examiner blinded to the experimental groups detected cell optical density labeled with Nrf2, and HO-1 under a 400× light microscope. The average optical density level of each slice was obtained by Image-Pro Plus 6.0 software (Media Cybernetics, USA)

### Western blot

Tissues in cortex were homogenized in RAPI lysis buffer (Beyotime, China), centrifuged at 14,000 g at 4°C for 30 min, and then the supernatants were collected as total proteins. To prepare the cytoplasmic and nuclear proteins, cells were lysed using a nuclear and cytoplasmic protein extraction kit (Beyotime, China) according to the manufacturer’s instructions. Equal amounts of protein extracts were separated by SDS-PAGE and transferred onto polyvinylidene difluoride membranes (Millipore, Bedford, MA, USA) by electrophoresis, and membranes were blocked with 5% nonfat milk in 0.1% Tween 20 in TBS for 1 h at room temperature. Blots were then incubated with the antibody for phosphorylated-Nrf2 (p-Nrf2), Nrf2, HO-1, VEGF, VEGRF2, histone H3, GAPDH, and β-actin (Santa Cruz Biotechnology, USA).

### ELISA analysis

VEGF and VEGFR2 level of ischemic brain (n=8 per group) on day 7 after MCAO was detected by using ELISA kits (R&D systems, USA) according to the manufacturer’s protocols.

### Primary cortical neuron culture

Cortical neurons were cultured using a modified method reported by Redmond et al. [19]. Briefly, Primary cortical neurons were generated from fetus of rats. Briefly, whole cerebral cortices were dissected, incubated for 15 min in 0.25% trypsin at 37°C and mechanically dissociated using Pasteur pipette. Cells were seeded at a density of 1.5×10^5^ cells per cm^2^ onto poly-D-lysine-coated 96- or 6-well plates and were kept in a humidified incubator in air with 5% CO_2_. After 24 h, the culture medium was changed to neurobasal medium supplemented with 2% B27. Medium replacement was performed every 3 days. After 15 days in culture, the cells form extensive axonal and dendritic networks and are ready for the experiments.

### Oxygen-glucose deprivation and reoxygenation

Cells seeded in 96-well plates were pretreated with CL at the indicated concentrations for 2 h. Then, to model ischemia-like conditions *in vitro*, primary cultured cortical neurons were exposed to transient OGD for 60 min. Finally, the neurons were incubated again in the incubator with 95% air and 5% CO_2_ with or without CL for an additional 24 h. OGD-induced cell death was quantified using the 3-[4,5-dimethylthiazol-2-yl]-2,5-diphenyltetrazolium bro-mide (MTT) assay. Lactate dehydrogenase (LDH) release was assessed by a commercially available kit (Jiancheng Biological Engineering Institute, Nanjing, China). Nrf2 Knockdown was achieved through transient transfection of primary cortical neuron cells by using Nrf2 siRNA and Lipofectamine RNAi MAX Reagent (Life Technologies, CA, USA).

### Statistical analysis

The statistical analyses were performed using SPSS 18.0 (SPSS Inc., Chicago, IL, USA). All of the values were presented as mean ± standard deviation (SD), except for the neurological deficit score, and differences between groups were compared with one-way ANOVA followed by followed by Fisher’ s post hoc test. Neurological deficit scores were expressed as the median with range. This data was analyzed by the Kruskal-Wallis test followed by the Mann-Whitney U test and Bonferroni post hoc correction. P<0.05 was regarded statistically significant.

## SUPPLEMENTARY MATERIALS FIGURE AND TABLE



## Abbreviations

CL: corilagin
FITC: fluorescein isothiocyanate
GPx: glutathione peroxidase
IR: ischemia-reperfusion
HE: hematoxylin and eosin
HO-1: heme oxygenase-1
Keap1: Kelch-like ECH-associated protein 1
MCAO: middle cerebral artery occlusion
MDA: malondialdehyde
MTT: 3-[4,5-dimethylthiazol-2-yl]-2,5-diphenyltetrazolium bromide
Nrf2: nuclear factor erythroid-2-related factor 2
OGD: oxygen and glucose deprivation
PBS: phosphate-buffered saline
p-Nrf2: phosphorylated-Nrf2
ROS: reactive oxygen species
SD: standard deviation
siRNA: small interfering RNA
SOD: superoxide dismutase
TTC: 2,3,5-triphenyltetrazolium chloride
TUNEL: terminal deoxynucleotidyl transferasedUTP nick end labeling
VEGF: vascular endothelial growth factor
VEGFR2: vascular endothelial growth factor receptor 2.
