# Supplementary material for: Antioxidant and pro-angiogenic effects of corilagin in rat cerebral ischemia via Nrf2 activation
Source: Oncotarget. 2017 Oct 24;8(70):114816–28. doi: 10.18632/oncotarget.22023 (PMC5777734; doi:10.18632/oncotarget.22023)
Supplement: Supplementary file 1 [file oncotarget-08-114816-s001.pdf]

Antioxidant and pro-angiogenic effects of corilagin in rat cerebral ischemia via Nrf2 activation

SUPPLEMENTARY MATERIALS

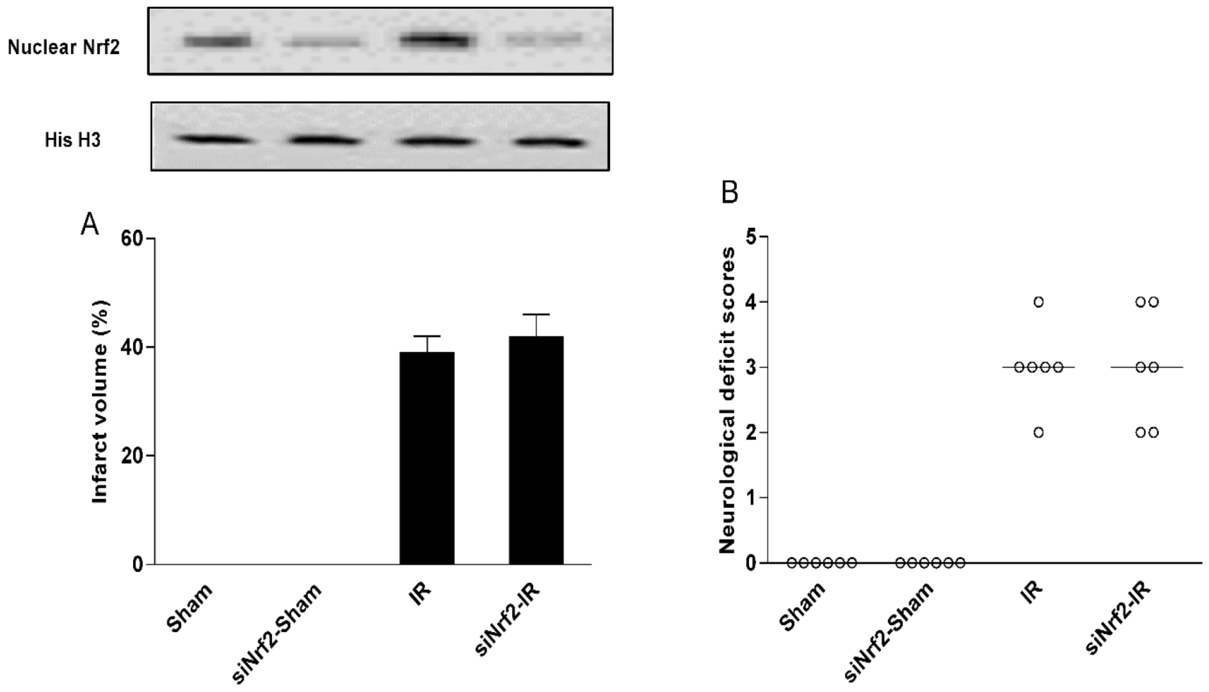

Supplementary Figure 1: Transfection of siRNA has no significant effects on brain injury and improves neurological outcome in normal and MCAO rats. Transfection efficiency was checked by Western analysis. **(A)** Quantitative analysis of infarct size and Nrf2 expression. Values are mean ± SD (n=5). **(B)** Neurological scores of animals in different groups. Data are presented as the median (range) (n = 5).

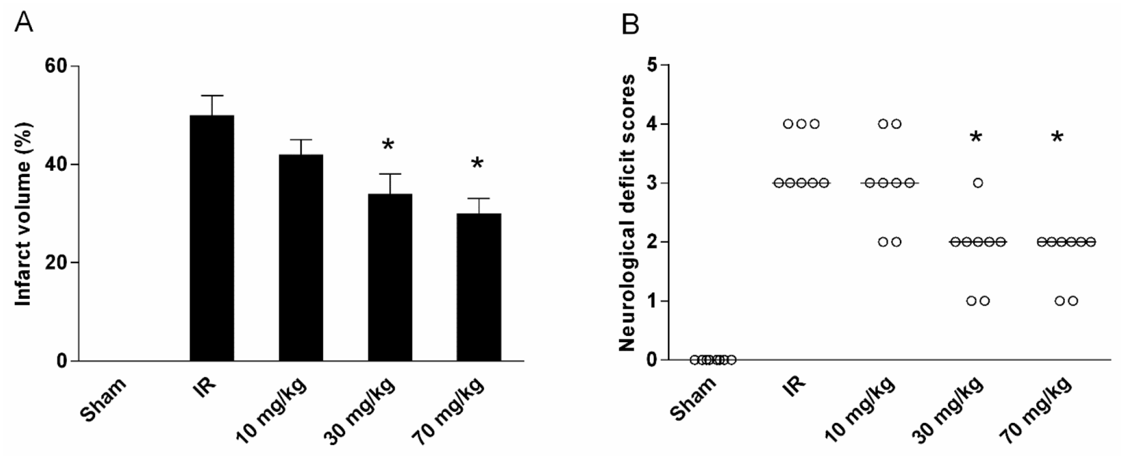

Supplementary Figure 2: With the treatment of CL, infarct volume **(A)** and neurological outcome **(B)** was greatly improved in a dose-dependent manner as compared with the vehicle group. *P<0.05 CL versus vehicle treated group. (n=5 animals for each group).

Supplementary Table 1: Physiologic parameters, mean ± SD (n=8)

	MAP (mm Hg)	pH	$PaCO_2$(mm Hg)	PaO_2(mm Hg)	Glu (mg/dl)
			Pre-ischemia		
Sham	121.3±5.2	7.30±0.12	40.4±3.6	148.6±12.2	143.3±7.5
IR	120.6± 5.1	7.41±0.17	41.6±3.4	144.5±13.5	133.5±6.2
IR-CL	114.1±6.4	7.33±0.15	39.5±3.7	147.5±15.6	137.1±8.7
Si-CL	121.6± 5.2	8.11±0.24	42.6±3.4	146.5±13.5	138.5±9.2
Con-CL	115.1±6.1	8.33±0.15	39.4±3.1	141.5±12.6	136.1±7.2
			15 min after reperfusion		
Sham	107.8±6.2	7.36±0.13	39.4±5.1	142.1±14.4	142.1±10.6
IR	110.5±4.3	7.38±0.16	39.1±4.7	134.0±12.8	145.5±8.9
IR-CL	106.1±5.3	7.40±0.05	40.2±2.5	132.7±15.3	141.6±8.7
Si-CL	105.5±4.5	7.238±0.16	36.1±4.1	131.0±12.8	143.1±8.1
Con-CL	102.1±5.1	8.40±0.07	38.2±3.5	132.7±15.3	141.6±8.6
			60 min after reperfusion		
Sham	107.8±5.6	7.41±0.29	38.2±3.9	140.0±16.4	142.9±8.3
IR	101.3±3.2	7.39±0.34	37.4±3.1	132.5±11.2	148.6±6.5
IR-CL	103.8±7.6	7.41±0.26	38.2±3.9	130.0±16.4	139.9±7.1
Si-CL	103.5±4.2	7.39±0.14	33.4±5.1	122.5±11.2	147.6±4.5
Con-CL	102.3±4.5	7.29±0.35	32.4±5.1	126.5±12.1	148.6±6.4

MAP, Mean arterial blood pressure; PaO_2, arterial oxygen tension; $PaCO_2$, arterial carbon dioxide tension; Glu, blood glucose.
